# The long-term impact of adverse childhood experiences on externalizing aggressiveness: sensitive periods during childhood and adolescence

**DOI:** 10.3389/frcha.2026.1765690

**Published:** 2026-04-10

**Authors:** Steffen Barra, Sina Groß, Anselm Crombach, Daniel Turner, Petra Retz-Junginger, Johannes Merscher, Wolfgang Retz

**Affiliations:** 1 Institute for Forensic Psychology and Psychiatry, Saarland University, Homburg, Germany; 2 Department of Psychology, Saarland University, Saarbruecken, Germany; 3 Department of Psychiatry and Psychotherapy, University Medical Center of the Johannes Gutenberg-University, Mainz, Germany

**Keywords:** age, aggression, maltreatment, prevention, violence

## Abstract

The association of adverse childhood experiences (ACEs) with the perpetration of aggressive behavior across the lifespan has repeatedly been demonstrated. However, whereas age-specific impacts of ACEs on neural and psychological development are discussed, little is known about sensitive periods during childhood and adolescence in which ACEs may exert the most severe effects on an individual's propensity for aggression. Thus, the present study retrospectively assessed ACEs during early childhood (up to 5 years), late childhood (6–11 years), and adolescence (12 years and above) in 204 adults and examined age-dependent relations to current externalizing aggressiveness. Although the general ACE-aggression link could be replicated, no clear evidence emerged for age-specific impacts. While the total ACE burden was highest during adolescence and only ACEs during adolescence were associated with increased adult aggressiveness, the strengths of the relationships between ACEs and aggressiveness did not differ significantly depending on the examined age groups. Nevertheless, our results underscore the need for future research to implement more sophisticated age-sensitive approaches to examine the association of ACEs with aggressive behavior as an important basis for the design of promising prevention and intervention measures.

## 1 Introduction

About every second individual in Western countries has experienced at least one stressful life event during childhood and adolescence, such as emotional neglect, domestic violence or sexual abuse, and many people are confronted with multiple of these so called adverse childhood experiences [ACEs; (1–3)]. The consequences of ACEs can be severe and diverse across the lifespan. Prior empirical findings emphasized long-term connections between ACEs and physical as well as mental illness, health risk factors, and psychosocial abnormalities [e.g., (3, 4)]. ACEs have been shown to increase the risk of internalizing problems like depression and anxiety [e.g., (5)], but also externalizing aggressive, violent, or criminal behavior [e.g., (4, 6, 7)]. There is strong evidence for a linear dose-response relationship between the number of cumulative ACEs and outcome severity [e.g., (1, 8, 9)]. Whether ACEs lead to internalizing or externalizing outcomes and, more specifically, to self-directed destructive thoughts, feelings, or behavior (internalizing aggressiveness) or respective tendencies directed toward other individuals (externalizing aggressiveness) may depend on a range of further influencing factors, such as gender, certain personality traits, cognitive and emotion processing strategies, (comorbid) psychiatric burden, and the types of ACEs as such (10–13).

Children and adolescents of all ages can be burdened by ACEs (2, 8). Although the prevalence and consequences of ACEs have received a lot of scientific attention overall, a specific consideration of the timing at which ACEs occur has, however, hardly been included in research so far. Yet, some studies have shown that the likelihood of experiencing certain ACEs varied across different age groups. For example, Teicher and Parigger (2) examined a sample of healthy young adults and identified an age-dependent variance in ACE severity for nonverbal emotional abuse by parents (continuous increase and peak at 14–18 years), physical abuse by parents (peak at 5–8 years), verbal abuse by parents (peak at 11–16 years), and emotional abuse by peers (peak at 10–14 years). Among adult psychiatric patients, reported ACEs peaked in frequency and severity at age 13 (8). In their recent publication on time specific ACEs in 185 young adults with a history of out-of-home placements, Meier and colleagues (14) found that parental neglect was highly prevalent all across childhood and adolescence (3–18 years of age), whereas other types of ACEs, including parental abuse, sexual abuse, and peer violence, tended to be most frequent in adolescence. In a study of 278 male adolescents convicted of sexual offences (15), each type of ACE was most common in late childhood (6–11 years) and, except for physical abuse, least common in early childhood (0–5 years). The few studies that have taken into account age specifics have also indicated that the timing and duration of ACE exposure can influence respective consequences, too (8, 14–18). For example, Schalinski et al. (8) found a dose-response relationship between the duration of ACE exposure and symptoms of severe mental illness in adult psychiatric patients. Among formerly out-of-home placed young adults, timing specific effects emerged as well, especially for the associations of early childhood parental abuse and adolescent peer violence with externalizing behavior problems (14). Thornberry and colleagues (16, 17) found that individuals who had only experienced abuse in early childhood (up to 5 years) had no long-term consequences. Those affected in late childhood (6–11 years) had a higher risk for only a few dysfunctional outcomes, including delinquency. In contrast, participants who only experienced abuse in their youth (12 years and above) or continuously during childhood and adolescence had an increased risk for a variety of negative consequences, such as delinquency, drug use, and alcohol-related problems. Barra et al. (15) operated with the same age groups when investigating the probability of criminal recidivism in juveniles who had sexually offended. A significant positive association was found between non-sexual recidivism and severe/cumulative ACEs (especially related to neglect), which was independent of the age at exposure. Sexual recidivism was significantly associated with sexual victimization in adolescence and time-independent neglectful experiences.

One explanatory approach regarding age specific impacts of ACEs on long-term dysfunctional consequences was proposed by researchers who emphasized stress-sensitive periods in brain development [e.g., (9, 18, 19)]: According to these scholars, brain development—while driven by genes—is also significantly influenced by experiences, especially in sensitive phases that may be particularly vulnerable for ACEs. Gene-environment interactions have been proposed in the context of ACEs and externalizing behavior problems [e.g., (20)] and research shows progress in the investigation of epigenetic modifications in this area [e.g., (21)]. A literature review highlighted the vast number of studies that were able to establish a connection between ACEs and changes in both brain structures and functions (9). When focusing on increased risk of externalizing aggressiveness as one potential maladaptive outcome of ACEs, neuroscientific studies have found potentially stress-related deviations in the morphology, functionality, and connections of certain brain areas related to aggressive behavior, e.g., regarding the prefrontal and orbitofrontal cortex, or the limbic system including the amygdala and hippocampus (22–25). For instance, in an fMRI study, orphanage children who had experienced institutional deprivation showed increased amygdala activity compared to the control group when performing an emotional face go/nogo task with fearful vs. neutral face stimuli (26). Increased amygdala response to emotional, especially threatening, faces was also found in other individuals with ACE histories (9). Further studies point to amygdala and hippocampus volume deviations indicating a dose-response relationship with the severity of ACE exposure (18, 19). However, the existing evidence is not conclusive as other research had failed to detect ACE related volume changes in relevant brain areas (27, 28). The divergent findings may be traced back to the assumption that certain brain areas are particularly sensitive to environmental influences during specific time periods (29). For example, Andersen et al. (18) found that the frequency of ACEs at the age of three to five years was associated with a significant reduction in the volume of the hippocampus; at the age of 11–13 years, the effect was marginally significant and non-existent in other age groups. No such connection could be found for the amygdala in this study, which is why the authors postulated that the amygdala would not have a specific measurable sensitive phase. However, other researchers found sensitive periods for the development of amygdala volume at age 10–11, and for right hippocampal volume at ages seven and 14 (19). Yet, it must not be neglected that these brain regions also show specific developmental trajectories regardless of ACEs (29, 30).

Overall, the current state of research indicates that there might be reason to assume that externalizing aggressiveness as a long-term consequence of ACEs may depend on the timing of ACE occurrence. However, there is a lack of respective studies investigating this assumption in more detail. This is particularly regrettable because such investigations could form the basis for the development of age-sensitive prevention and intervention approaches aimed at reducing the occurrence of ACEs at particular time periods during childhood and adolescence and decreasing their impact on the risk of aggressive outcomes across the lifespan; thus, contributing not only to an individual's healthy development but also to the protection of society from potential victimization.

To address this research need, the present study investigated the possibility to retrospectively identify age periods in which ACEs had the strongest impact on adult externalizing aggressiveness. Based on the abovementioned previous but inconclusive research, it was expected that (a) the probability of high externalizing aggressiveness in adulthood would be positively related to the global (age-independent) severity of ACEs, (b) the ACE load would differ among early childhood (up to 5 years), late childhood (6–11 years), and adolescence (12 years and above), and (c) that the strengths of the associations of ACEs with adult externalizing aggressiveness would vary depending on the age at ACE occurrence.

## 2 Materials and methods

### 2.1 Procedure and sample

The present study was part of a broader project implemented by the Institute for Forensic Psychology and Psychiatry, Saarland University, Homburg, in cooperation with the Forensic Psychiatry and Psychotherapy Section of the Johannes Gutenberg-University's Medical Center, Mainz, which aimed at investigating clinically and forensically relevant risk factors in adults [e.g., (31, 32)]. Within the general clinical procedures for individuals referred to the abovementioned institutions for forensic evaluation/therapy (e.g., assessment regarding criminal responsibility or recidivism risk if they had been accused of a crime, or psychotherapeutic treatment after completion of a prison sentence) or (non-forensic) clinical assessment (e.g., regarding adult attention-deficit/hyperactivity disorder; ADHD), a set of self-report questionnaires was to be completed, including those considered for this study. The patients themselves could decide whether their information could be used anonymously for research purposes. In addition, psychology and medical students who were affiliated with the above facilities invited acquaintances for their participation in the study, who were not referred to the abovementioned institutions. Besides the minimum age of 18 years and sufficient German language skills, no specific inclusion or exclusion criteria were defined. Thus, we were able to assess a heterogeneous sample with forensic, clinical, and non-forensic/non-clinical background aiming at achieving an appropriate variance in our variables of interest. Prospective participants were told that participation was voluntary and unpaid, and informed consent was obtained. The questionnaire set could be filled out on site or online (https://www.soscisurvey.de). Study procedures were based on the ethical standards of the Declaration of Helsinki. The ethics committee of the Medical Chamber of Saarland, Germany, approved the research project (protocol code: 58/22).

A total of 405 individuals were assessed between 11 November 2021 and 26 June 2024. Of those, 15 had not agreed to have their data analyzed for research purposes. Moreover, 75 had not completed the questionnaires relevant to the present study (see below). A further 111 cases were excluded due to incomplete information on the age at ACE occurrence. Thus, data of 204 subjects could eventually be considered. The participants' age at assessment ranged between 18 and 75 years (M = 33.22 years, SD = 12.18 years). The sample showed a balanced distribution regarding male (*n* = 92, 45.1%) and female (*n* = 109, 53.4%) gender identification, with three participants describing themselves as diverse (*n* = 3, 1.5%). As highest level of completed education, about one-third of the sample (*n* = 69, 33.8%) reported having a German high school diploma (“Abitur”). Another 26.9% (*n* = 55) had a university (Bachelor's or Master's) degree, three a doctoral degree (1.5%). Whereas 13.2% (*n* = 27) reported having a secondary school certificate (“Haupt- or Realschulabschluss”), about one-fifth of the sample had a vocational school qualification (*n* = 39, 19.2%). Eight participants (3.9%) had a special education or no school leaving certificate and another three (1.5%) indicated some different, not further specified qualification. Forty-three individuals (21.1%) had been referred to forensic evaluation/therapy, 54 (26.5%) for clinical assessment, and 107 (52.4%) had been recruited by personal addressing of students.

### 2.2 Measures

#### 2.2.1 Adverse childhood experiences

ACEs were assessed by the German version of the Maltreatment and Abuse Chronology of Exposure scale [MACE-X; (2, 33)]. The questionnaire covers 10 types of ACEs by a total of 75 items: parental verbal abuse, parental non-verbal emotional abuse, parental physical abuse, emotional neglect, physical neglect, witnessing violence towards/between parents, witnessing violence towards siblings, emotional abuse by peers, physical abuse by peers, and sexual abuse. After indicating on each item whether the described ACE had occurred at any point in time during childhood and adolescence (yes/no), the specific age at occurrence can be reported on a scale of one to 18. A growing body of research points to the psychometric value and applicability of the MACE-X in various samples (2, 8, 14, 31–33).

There are different possibilities to transfer item scores into higher-level scales, depending on whether the severity or the multiplicity (number of different types) of ACEs is focused. For the present study, severity measures were conducted using the linear interpolation approach proposed by the developers of the German version of the MACE-X (33): Because the ten subscales contain different numbers of items (between 4 and 10), each subscale score is first multiplied by the quotient of 10 and the number of items, so that each subscale can reach a maximum value of 10. Thus, the global ACE severity (sum) score can range between 0 and 100, with higher values indicating increased ACE load. In addition to a global ACE severity score that represented an individual's age-independent ACE load, we investigated time-specific ACE severity at three age periods that had been used in prior research (15–17): early childhood (up to 5 years), late childhood (6–11 years), and adolescence (12 years and above).

#### 2.2.2 Externalizing aggressiveness

Externalizing aggressiveness was assessed by the German Short Questionnaire for the Assessment of Aggressiveness Factors [K-FAF; (34)]. This self-report measure includes a total of 49 items to be rated on a six-point Likert scale between “strongly disagree” to “strongly agree”, which refer to different aspects of aggressiveness: spontaneous aggressiveness, reactive aggressiveness, excitability, self-aggressiveness, and aggression inhibition. The first three dimensions add up to an overall sum score that describes an individual's willingness to show outward-directed aggression, thus used to operationalize externalizing aggressiveness. Good psychometric properties were reported for the K-FAF by former scholars (34, 35). Internal consistency of the sum score in the current sample (Cronbach's *α* = .93) was comparable to findings in the German norm sample (Cronbach's *α* = .89) which consisted of aggressive/delinquent and healthy/non-delinquent subjects. For the present study, the individuals' externalizing aggressiveness scores were transformed into T-values originating from this norm sample as reported in the test manual (34). The current sample was then divided into individuals with or without above-average, concerning externalizing aggressiveness (*T* ≥ 60 or *T* < 60, respectively).

### 2.3 Statistical analyses

Statistical analyses were conducted in IBM SPSS Statistics Version 30 for Windows. Age at assessment and gender were included as covariates to account for potential confounding. Group differences in variable distributions were analyzed by Chi²-tests, (M)ANOVAS, and *t*-tests. Relational structures among variables were investigated by Pearson correlations. Associations of ACEs with externalizing aggressiveness were examined by binary regression models. The general significance level was set to *α* = .05. Internal consistency (Cronbach's *α*) was interpreted as good with *α* ≥ 0.80 and excellent with *α* ≥ 0.90 (36). Correlation coefficients of |*r*| = 0.10 pointed to small, |*r*| = 0.30 to medium, and |*r*| = 0.50 to large correlations; thresholds for the effect size Cohen's d were |*d*| = 0.2 for small, |*d*| = 0.5 for medium, and |*d*| = 0.8 for large effects, and the effect size partial eta squared (*η*^2^_p_) represented a small effect with *η*^2^_p_ ≤ 0.01, a moderate effect with *η*^2^_p_ = 0.06, and a large effect with *η*^2^_p_ = 0.14 (37, 38).

## 3 Results

### 3.1 Descriptives

The mean global (age-independent) ACE severity score in the total sample was 17.76 (SD = 15.60, range = 0–66.33). Slight, but not statistically significant gender differences emerged, *F*(2, 201) = 1.52, *p* = .221, *η*^2^_p_ = .015 (males: M = 15.88, SD = 13.32; females: M = 19.49, SD = 17.32; diverse: M = 12.44, SD = 7.50). Age at assessment and global ACE severity were not significantly correlated (r = -0.58, *p* = .412). An overview of the individual ACE types that have been reported can be found in the supplements (Supplementary Table 1). Externalizing aggressiveness raw scores averaged at 40.36 (SD = 25.70, range = 3.00–157.00), T-values ranged between 27.00 and 80.00 (M = 47.97, SD = 14.05). About one-fifth of the participants (*n* = 43, 21.1%) showed T-scores ≥ 60, with a slim majority being male (*n* = 25, 58.1%), although gender distribution did not significantly deviate from an expected equal distribution, *χ*²(2) = 4.22, *p* = .121. On average, subjects with concerning externalizing aggressiveness were younger than those without [M = 30.53 years, SD = 8.42 years vs. M = 33.96 years, SD = 12.97 years; t(104.92) = 2.10, *p* = .038, d = .28].

### 3.2 Hypothesis testing

**Figure 1:**
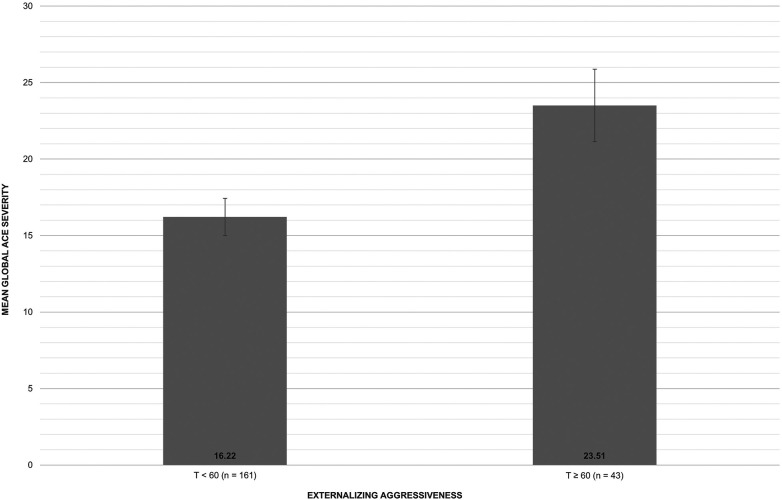
Global ACE severity in individuals without and with concerning externalizing aggressiveness.

Under statistical control of age at assessment and gender, a significant positive correlation was found between global ACE severity and dimensional externalizing aggressiveness, *r* = .23, *p* < .001. A linear regression model confirmed this relationship. Adding the global ACE severity score significantly improved a model including age at assessment and gender (ΔR² = 0.05, *p* < .001). This model [F(3, 197) = 8.40, *p* < .001] explained about 11.3% of the variance with a significant association of the global ACE severity score with dimensional externalizing aggressiveness [b = 0.21, 95% CI = (0.09, 0.33), *p* < .001, *β* = 0.23]. In line with this finding, individuals with concerning externalizing aggressiveness scores (T ≥ 60) showed higher global ACE severity (M = 23.51, SD = 15.54) than those without (M = 16.22, SD = 15.29), F(1,201) = 7.96, *p* = .005, *η*^2*p*^ = .04 (see Figure 1). Binary logistic regression showed that global ACE severity was significantly associated with the assignment to the concerning externalizing aggressiveness group (B = 0.03, *p* = .005, OR = 1.03, 95% CI [1.01, 1.06].

**Figure 2:**
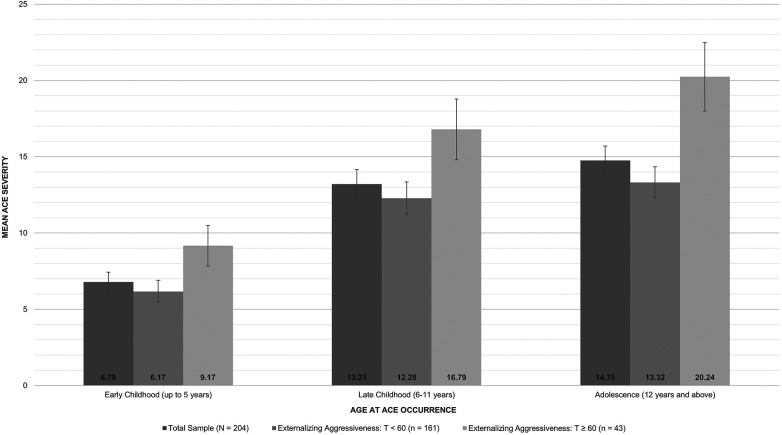
Age-dependent ACE severity in the total sample and separated for individuals without and with concerning externalizing aggressiveness.

The total sample displayed the highest ACE severity in adolescence (M = 14.75, SD = 13.55, range = 0.00–62.67), followed by late childhood (M = 13.21, SD = 13.56, range = 0.00–58.58), and early childhood (M = 6.79, SD = 9.21, range = 0.00–53.75); see Figure 2. Partial correlations among early childhood, late childhood, and adolescent ACE severity scores (controlled for age at assessment and gender) were high (early/late childhood: r = .85, early childhood/adolescence: r = .77, late childhood/adolescence: r = .88; all *p* < .001). However, overall mean differences in ACE severity between age periods were significant, F(1.81, 357.26) = 14.77, *p* < .001, *η*^2*p*^ = .07. Simple contrasts showed relevant deviations between early childhood and both late childhood, F(1, 197) = 14.44, *p* < .001, *η*^2*p*^ = .07, and adolescence, F(1, 197) = 21.60, *p* < .001, *η*^2^_p_ = .10, but not between late childhood and adolescence, F(1, 197) = 3.33, p = .069, *η*^2^_p_ = .02.

Figure 2 also illustrates that individuals with concerning externalizing aggressiveness reported greater ACE loads than those without at each age period (early childhood: M = 9,17, SD = 8.62 vs. M = 6.17, SD = 9.28); late childhood: M = 16.79, SD = 12.91 vs. M = 12.28, SD = 13.61; adolescence: M = 20.24, SD = 14.55 vs. M = 13.32, SD = 12.94). The group differences were significant when age and gender were taken into account, F(3, 194) = 3.93, *p* = .009, *η*^2*p*^ = .06 [early childhood: F(1,196) = 5.10, *p* = .025, *η*^2^_p_ = .03; late childhood: F(1,196) = 4.19, *p* = .042, *η*^2^_p_ = .02; adolescence: F(1,196) = 9.37, *p* = .003, *η*^2^_p_ = .05]. Despite the high correlations among age specific ACE severities, multicollinearity statistics allowed for further regression analyses (all VIF <7, no tolerance value <.01). Eventually, binary logistic regression models showed that ACE severity at each age period was associated with concerning externalizing aggressiveness in adulthood when analyzed separately, thus without considering the other age periods (Table 1). When age periods were jointly taken into account within one logistic regression model, however, only ACE severity in adolescence remained associated (see Table 2). To compare effect sizes, we ran three additional regression models including difference and sum variables for (1) early and late childhood ACE severity scores, (2) early childhood and adolescent ACE severity scores, and (3) late childhood and adolescent severity scores, each controlled for age and gender. A significant regression coefficient for the difference variable would indicate a meaningful difference in the strengths of the accordant predictors (39). However, none of these differences were significant (see supplements, Supplementary Table 2).

**Table 1:** Binary logistic regression models for the associations of concerning externalizing aggressiveness with single age-dependent ACE severities.

ACE Severity	Concerning externalizing aggressiveness				
				95% CI	
	*B*	*p*	*OR*	*LL*	*UL*
Early Childhood (up to 5 years)	0.04	.025	1.04	1.01	1.08
Late Childhood (6–11 years)	0.03	.036	1.03	1.00	1.05
Adolescence (12 years and above)	0.04	.003	1.04	1.01	1.07

*N* = 204. All analyses controlled for age at assessment and gender. LL, lower limit; UL, upper limit.

**Table 2:** Binary logistic regression models for the associations of concerning externalizing aggressiveness with joint consideration of age-dependent ACE severities.

ACE Severity	Concerning externalizing aggressiveness				
				95% CI	
	*B*	*p*	*OR*	*LL*	*UL*
Early Childhood (up to 5 years)	0.03	.469	1.03	0.96	1.10
Late Childhood (6–11 years)	−0.04	.195	0.96	0.90	1.02
Adolescence (12 years and above)	0.06	.016	1.07	1.01	1.12

*N* = 204. All analyses controlled for age at assessment and gender. LL, lower limit; UL, upper limit.

## 4 Discussion

The present study investigated the relationship between ACEs and adult externalizing aggressiveness by taking into account the timing of ACE occurrence. Regardless of the age at assessment and gender, individuals with elevated scores on externalizing aggressiveness reported higher global (age-independent) ACE severity than those without, and global ACE severity was positively associated with increased external aggressiveness. These results confirmed our first hypothesis and underline once again that facing stressful experiences during childhood and adolescence can have long-term consequences. Supporting theoretical models regarding the ACE-aggression link, our analyses indicated a dose-response relationship between ACEs and concerning adult externalizing aggressiveness (3, 4, 6, 40, 41). The repeatedly documented association of ACEs with aggression emphasizes that besides possible genetic dispositions, early environmental influences are crucial for the explanation of aggressive behavior, not only in the short term, but also in the long term. ACEs may shape an individual's neural, cognitive, and emotional development and foster maladaptive behavioral scripts (9, 18, 19, 42–44), increasing the risk of engaging in aggressive conduct towards others.

Although these developmental processes may be dependent on specific sensitive periods, little is known so far about how the timing of ACEs could influence later aggressive tendencies. Thus, the current study also differentiated for ACE severity at early childhood (up to 5 years), late childhood (6–11 years), and adolescence (12 years and above), as well as their links to adult externalizing aggressiveness. Consistent with our assumption, ACE severities differed depending on the age at ACE occurrence. The lowest ACE severity was found for the early childhood period, whereas the highest ACE severity was reported in adolescence. However, whereas early childhood ACE severity was clearly distinguishable from the later age periods, no significant difference emerged between late childhood and adolescence. These results were evident both in the overall sample and when distinguishing between individuals with and without concerning externalizing aggressiveness. The increased risk of ACE victimization with older age may be attributed to both individual and interpersonal challenges during puberty, but must not be interpreted without accounting for the possibility of reporting bias in our age-mixed sample (45). However, since largely in line with previous research (2, 8, 14), these time-dependent differences in ACE severity point to the need for age-sensitive preventive measures that address the specific demands of young individuals and their social contacts during these pivotal developmental stages.

At each of the examined age-periods, individuals with concerning externalizing aggressiveness showed higher ACE severity than those without, illustrating that the ACE-aggression link may be a rather universal than age-dependent phenomenon. Nonetheless, the largest group difference emerged during adolescence, and—when controlling for the other age-periods—adolescent ACE severity remained significantly associated with increased risk for concerning externalizing aggressiveness in adulthood, fitting with findings of prior scholars using comparable age-periods (15–17). From an evolutionary perspective [e.g., (46)], threatening experiences during adolescence, a sensitive phase for various individual and social change and adaption processes, may be of specific relevance for the acquisition of self-defending but also goal-oriented aggressive behaviors to gain dominance and status; (repeatedly) accompanied by experiences of success, these behaviors may foster the solidification of long-term aggressive scripts. Yet, the considerable intercorrelations of the examined age-dependent ACE severity scores showed that ACE loads may often be durable across childhood and adolescence, complicating the analysis and topic-related hierarchization of a-priori defined age periods. Moreover, our additional analyses question any clear timing-specific influence and contradict the confirmation of our third hypothesis.

The present results must not be interpreted without considering certain limitations. First, although aiming at examining a heterogeneous sample with forensic, clinical, and non-forensic/non-clinical backgrounds, data were assessed by two institutions in Germany only, which limits the generalizability of the results and indicates the need for follow-up research in further samples. Second, diagnostic criteria for certain psychiatric disorders were not evaluated in the present study, although this could have provided relevant information to understand the ACE-aggression link in more detail. Third, data relied on the participants' self-reports only, which might have been influenced by social desirability and memory bias (45, 47, 48); for instance, the recency effect [e.g., (49)] may have left ACEs faced in adolescence more accessible to the participants than earlier experiences, which should also be taken into account when interpreting their associations with externalizing aggressiveness. Furthermore, the 75-item version of the German MACE may have the benefit of assessing a wide range and the timing of ACEs, but its length can challenge a respondent's motivation to give detailed information, especially when included in an even larger questionnaire set. This might have been one potential reason for the lack of data on the age at ACE occurrence in more than 100 individuals. The use of shorter versions of the MACE with 20 or 40 items, preferable in an interview format (50, 51), could counteract this limitation in future studies. In addition, we were only able to assess the presence of ACEs but not the subjective burden an individual may have associated with these experiences, although prior research has emphasized that the latter seems to be of considerable relevance in the context of aggression [e.g., (52)]. Moreover, the predefinition of age-periods regarding ACE timing gives rise to critical discussion. Although age at ACE occurrence was categorized based on previous research for reasons of comparability and facilitation (15–17), these age-periods may not have been sufficiently suitable to reflect and differentiate underlying developmental processes, as, for instance, proposed by previous brain research (18). Although it is common to use ACE sum scores to represent overall ACE severity, time-dependent effects may also arise for specific types of ACEs, and outcomes may be influenced by their temporal sequence and, particularly, chronicity [e.g., (15)]. Other scholars have recently implemented alternative analytic approaches to investigate the types and timing of ACEs as well as their occurrence patterns, some even without the need to build presumed age periods [e.g., (14, 15)], which should inspire further research examining age-dependent ACE effects on later aggressive tendencies. Eventually, the present cross-sectional design could not include further influencing factors, such as mental health conditions or treatment effects, which underlines the need for future longitudinal approaches with control of other relevant variables.

Despite these limitations, the present study contributes to the scientific field that focuses on the prevention of aggression and violence, as it delivers further evidence for the ACE-aggression link and draws attention to a differentiated view of this connection with regard to age-related effects. These findings may inspire future research as well as prevention and intervention aimed at reducing the risk of life-time aggressive conduct, but also victimization during childhood and adolescence. Preventive measures should be sensitive for different risks of ACE occurrence between younger and older minors, and interventions with individuals who show elevated rates of externalizing aggressiveness may implement life-course oriented approaches addressing ACEs, such as Forensic Offender Rehabilitation Narrative Exposure Therapy [FORNET; e.g., (53)].

## Data Availability

The data analyzed in this study are subject to the following licenses/restrictions: The datasets presented in this article are not readily available because of the specific confidentiality of the assessed clinical and forensic information. Scientists wishing to use them for non-commercial purposes are kindly asked to contact the present authors to frame individual agreements. Requests to access these datasets should be directed to SB, steffen.barra@uni-saarland.de.

## Supplementary material

SUPPLEMENTARY TABLE S1 Prevalence of global and time-specific ACE types.

SUPPLEMENTARY TABLE S2 Binary logistic regression models for effect size comparison in the prediction of concerning externalizing aggressiveness by joint consideration of age-dependent ACE severities.
