# Supplementary material for: The long-term impact of adverse childhood experiences on externalizing aggressiveness: sensitive periods during childhood and adolescence
Source: Front Child Adolesc Psychiatry. 2026 Apr 10;5:1765690. doi: 10.3389/frcha.2026.1765690 (PMC13106129; doi:10.3389/frcha.2026.1765690)
Supplement: SUPPLEMENTARY TABLE S1 — Prevalence of global and time-specific ACE types. [file Table1.docx]

Table S1. Prevalence of global and time-specific ACE types.

| Type of ACE | Timing of occurrence | | | | | | | |
| --- | --- | --- | --- | --- | --- | --- | --- | --- |
|  | Global | | Early Childhood  (up to 5 years) | | Late Childhood  (6-11 years) | | Adolescence  (12 years and above) | |
|  | *n* | *%* | *n* | *%* | *n* | *%* | *n* | *%* |
| parental verbal abuse | 111 | 54.4 | 42 | 20.6 | 90 | 44.1 | 105 | 51.5 |
| parental non-verbal emotional abuse | 116 | 56.9 | 52 | 25.5 | 96 | 47.1 | 108 | 52.9 |
| parental physical abuse | 90 | 44.1 | 42 | 20.6 | 77 | 37.7 | 60 | 29.4 |
| emotional neglect | 150 | 73.5 | 142 | 69.6 | 145 | 71.1 | 144 | 70.6 |
| physical neglect | 90 | 44.1 | 64 | 31.4 | 73 | 35.8 | 78 | 38.2 |
| witnessing violence towards/between parents | 21 | 10.3 | 11 | 5.4 | 15 | 7.4 | 10 | 4.9 |
| witnessing violence towards siblings | 35 | 17.2 | 14 | 6.9 | 25 | 12.3 | 23 | 11.3 |
| emotional abuse by peers | 121 | 59.3 | 13 | 6.4 | 89 | 43.6 | 110 | 53.9 |
| physical abuse by peers | 45 | 22.1 | 3 | 1.5 | 29 | 14.2 | 36 | 17.6 |
| sexual abuse | 44 | 21.6 | 10 | 4.9 | 16 | 7.8 | 34 | 16.7 |
| *Notes*: *N* = 204. Type of ACE was counted if at least one associated item had been affirmed.  if at least one associated item was answered affirmatively. | | | | | | | | |

Table S2. Binary logistic regression models for effect size comparison in the prediction of concerning externalizing aggressiveness by joint consideration of age-dependent ACE severities.

|  | ACE Severity Difference | Concerning externalizing aggressiveness | | | | |
| --- | --- | --- | --- | --- | --- | --- |
|  |  |  |  |  | 95% CI | |
|  |  | *B* | *p* | *OR* | *LL* | *UL* |
| Model 1 | Early Childhood  (up to 5 years) – Late Childhood (6-11 years) | 0.01 | .711 | 1.01 | 0.96 | 1.07 |
| Model 2 | Early Childhood  (up to 5 years) – Adolescence (12 years and above) | -0.02 | .335 | 0.98 | 0.94 | 1.02 |
| Model 3 | Late Childhood (6-11 years) - Adolescence  (12 years and above) | -0.05 | .067 | 0.96 | 0.91 | 1.00 |
|  | *Notes. N* = 204. All analyses controlled for age at assessment and gender as well as respective age-dependent sum variables. *LL = lower limit, UL = upper limit.* | | | | | |
